# Camp Diarrhœa and Dysentery

**Published:** 1875-04

**Authors:** T. M. Johnson


ART III.—*Camp Diarrhœa and Dysentery* By T. M. JOHNSON, M. D. Read before the Buffalo Medical Association, January 5, 1875.

*Mr. President and Gentlemen of the Association.*—It may perhaps seem at first glance that the subject of camp diarrhœa and dysentery would be chiefly interesting to medical men connected with military service.

But the general prevalence of diarrhœa and dysentery to a greater or less degree throughout our whole country, and their sameness in causation, course, pathology and treatment, whether seen in military or civil practice, render them of interest to every practitioner of medicine. We speak of two diseases conjointly, because their causation, course and pathology, especially in their chronic form, are so similar, and because they so frequently prevail together. In many instances, indeed, it is difficult to decide, during the progress of the case, whether we have a case of chronic dysentery or one of those cases of chronic diarrhœa that so closely resemble it.

They have always been severe scourges of all wars of which we have any medical history. During our late war of the rebellion they constituted on the average more than one-fourth of all the cases of disease reported. The annual number of cases reported, alike in the Union and Confederate armies, was greater than three-fourths the mean strength of those armies, and with a mortality second only to camp fever. The ratio of cases was seven hundred and sixty-five per one thousand the first year, and eight hundred and fifty-two per thousand the second year. The mortality being at the rate of twelve and one-third per one thousand. In searching out the causes of these diseases their geography is of interest and shows that they are endemic in all parts of the United States. Their causes exist to a greater or less degree in all localities, some districts being only slightly subject to their development, while others develop them in greater number, severity and fatality.

Forry, in discussing the mortality of the U. S. army twenty-five years ago, showed that the proportion of deaths to cases of these diseases, at the posts in the northern division of the States, was one in six hundred and sixty-five; while in the southern division it was one in one hundred and forty-one, thus showing the mortality about four and a half times as great in the southern division as in the northern. These facts repeat themselves in the history of the great war of the rebellion. During the year 1863 the ratio per thousand of mean strength dying of these diseases was, in the

Atlantic or Eastern region, 8.80, in the Central region, which takes in the Mississippi Valley, 23.49, and in the Pacific region only, 80.

These contrasts prove conclusively that independently of causes incident to camp life there are certain climatic or geographic causes which largely influence the frequency and fatality of these diseases. Marsh miasma and the causes which produce intermittent fever greatly augment cases and mortality. In the marshy districts of the western and southwestern States the cases are more numerous and fatal than in the less malarial districts of the Atlantic and Pacific regions. The troops operating in the west were in the main as well clothed, fed and cared for as were the troops of the east, and their vastly greater mortality is attributable almost entirely to climatic or geographic causes. A further and more definite illustration of this may be found in the reports of the Military General Hospitals for the year 1863. During that year the proportion of deaths to cases was, in the hospitals of the New England States, one in forty-nine. In those of New York City and State, and of Newark, N. J., one in nineteen. In those of Pennsylvania and Delaware, one in fifteen. In those in and about Washington and Baltimore, one in eleven. In those along the coast of the Carolinas, one in seven. At Nashville and Memphis, one in five. At New Orleans and Baton Rouge, La., one in four. The highest rate of mortality, however, was not at a point the furthest south, but at Cairo, Illinois, situated on a peninsula formed by the junction of the Mississippi and Ohio rivers, where most intense malarial influences prevail. The most prominent of the special causes which tend to the production of these diseases are, defective diet, exposure to rapid radiation of animal heat, scurvy, crowd-poisoning, depressing mental emotions, the depressing and relaxing influence of other diseases, and last, though not least, *previous attacks.* Unlike camp fever and many other diseases, these fluxes have a strong tendency to repeat themselves. For a long time after the apparent cure of a chronic diarrhœa, it is easily and frequently brought on again by improper diet, undue exposure or fatigue.

Veteranship is no protection against it, and statistics of both

Union and Confederate medical departments show that the longer a soldier is exposed to the vicissitudes of camp life the more likely is he to suffer from these diseases. Crowd-poison, filth and insufficiency of food existed in so small a degree in the Union armies as to be of no value as furnishing facts for scientific deductions. Unfortunately, however, the terrible statistics of the Confederate prison, particularly that at Andersonville, Ga., show that the number of cases and mortality were greatly augmented by these causes.

From a report made by Dr. Joseph Jones, C. S. A., to the Confederate Surgeon General, we learn that, during the six months from March to August, inclusive, in 1864, there occurred 4529 deaths from diarrhœa and dysentery among less than 40,000 Federal prisoners confined there. And we are given to understand from the same source that later in the same year the mortality was greatly increased. This terrible mortality was largely due to the three causes above mentioned. Statistics, however, from all field, hospital and prison reports combined, fail to give the true mortality from these diseases, for thousands affected with them were discharged or sent home on furlough from each of these three above sources, who found relief, in many instances, a welcome relief in death. Those thousands, of course, do not appear in reports or statistics.

These fluxes are oftentimes, in the outset, caused by over repletion, or by food or drink that is unwholesome. They are also frequently preceded by constipation, and are ushered in by frequent painless dejections from the bowels accompanied with some anorexia and a slight malaise. The tongue is more or less furred, with often foul breath, and in some cases nausea, with or without vomiting. As the disease progresses the dejections become more frequent and are preceded by and accompanied with griping pains, which cease after the stool, and return again at longer or shorter intervals. In many cases there is a marked periodicity in these particulars, the after part of the day and fore part of the night being passed in comparative quiet until near daylight, when the griping stools recur and follow each other with frequency until about mid-day, when they again subside or come on much less frequently. This periodicity does not, however, present the peculiarities nor partake of the nature of an intermittent, nor is it curable by quinine. The discharges by stool, while they are always fluid and vitiated, exhibit considerable variety in appearance. In the same patient they vary from hour to hour ; sometimes being like pea soup, again like soap suds, and again containing undigested food, and exhibiting many shades of color, black and tar-like in appearance, green and gelatinous, and brown and fecal. In severe, long continued cases, they contain sheds of exuviæ, bloody mucous, and muco-purulent matter. During this time the constitutional symptoms have become of the gravest character. The patient has become more and more emaciated and debilitated, his skin has assumed a dirty, sallow hue, and a harsh, dry feel. The palm of his hands are hot and dry, the countenance is anxious. The mind is irritable, and the patient peevish, taciturn or morose. The tongue is red and dry, the pulse is small, feeble and quick. The eyes and cheek bones are prominent. Dirty, brown patches makes their appearance upon the surface, and the skin emits a sickly, offensive odor, while emaciation is going on rapidly. In some cases there is febrile action, but not well marked, and at the last well marked hectic. In many, long continued cases, there is œdema of the face, abdomen or limbs, or all of these. Many of the more prolonged cases continue for weeks or months and finally die from exhaustion. The pathology in all the various chronic forms of these fluxes is essentially the same, differing only in degree as found in the various stages of the disease. There are preserved in the Medical Museum, at Washington, more than two hundred specimens of sections of intestine illustrative of the pathology of diarrhœa and dysentery. The subjects from which they were taken had generally died of other diseases or gunshot wounds. These specimens are divided into five different groups. The first group contains examples of follicular ulceration of the colon, presenting all the transition forms of simple enlargement of the solitary folicles, the rupture of the same, and the formation of small, "punched out" ulcers. The colon is generally more or less thickened, the thickening sometimes amounting to a quarter of an inch. In a few cases in which the solitary folicles were simply enlarged, without ulcerations, the intestine was seldom thickened, was usually normal in color, but was sometimes slate or ash-colored, and sometimes presented congested patches. Sometimes the enlarged solitary folicles and the glands of Lieberkuhn were the seat of pigment deposits. In the more serious cases of diarrhœa the colon was more or less thickened, and presented the punched out ulcers which had originated in the enlarged solitary folicles. At this stage of the disease the colon was seldom normal in color. Sometimes it was red, reddish-brown or reddish-black. At other times greenish, slate or ash-color. The ulcers sometimes contained mucous and sometimes pus. The small intestine was seldom involved in this class of cases. The second group contains specimens illustrating cases in which the follicular ulcers have extended until, in some instances, a large part of the mucous membrance of the colon is destroyed. The follicular ulcers extend by burrowing in the sub-mucous connective tissue, and in this way frequently connect with eacn other. The mucous layer is frequently undermined by this burrowing process and perishes by sloughing. The second group of cases seem to be only a more advanced stage of the disease shown in the first. The third group of cases present more or less ulceration of bowel similar to those seen in the first and second, but in addition, the diseased surface is more or less coated with a greenish-yellow exudation similar to that observed in the air passages in diphtheria. This condition is seen in acute cases of dysentery, supervening upon chronic cases of diarrhœa of long standing. The fourth group includes those cases in which the *small*, as well as the large intestine is involved. Many of these are cases in which there has been complications of camp fever with diarrhœa, and the lesions of the small intestine are similar to those of the former disease. Besides these cases, however, there are several specimens in which, from the character of the lesion, it must be inferred that the affection of the small intestine is an extension of the disease upward from the colon. In such cases the ileum is thickened, the thickening being greater near the ileocæcal valve, and it presents ulcers similar to those of the colon, that appear to have arisen from the solitary folicles, and not from Peyer's glands.

The fifth group is composed of specimens illustrating tubercular ulceration of the bowels. The ulcers occur more frequently in the small intestine. Both small and large intestine, however, are often affected. This class of patients have tubercles in the lungs. Besides the peculiar tubercular ulcers of this class, the ulcers described in the four other groups appear in conjunction with them, so that they are like the other cases with the tubercular ulcer added.

It will be seen by the above illustrations that the seat of the initiation and inflammation is in the first place in the mucous coat, most frequently of the colon, less frequently of the small intestine, and that the point of origin of the ulcer is in the solitary glands of this region. These ulcers are also sometimes attended by metastatic abscess of the liver, and often by diphtheritic exudation in the mouth and air passages.

The treatment of the acute form of these diseases, which came I think to be almost universally adopted, was to give in the outset liberally of cathartic medicine. Sulphate of magnesia was I think the favorite; the favorite form and dose being an ounce of the saturated solution to which was added five to ten drops of aromatic sulphuric acid. Castor oil alone, or combined with tincture opii, was perhaps second in popularity. Whether the case had been preceded by constipation or not, experience proved that it was the best and most speedy method of cure, to free the bowels from all irritant, relieve the portal circulation and prepare the way for the successful administration of opiates, either alone, or combined with astringents.

Many cases, a large majority, indeed, were cured in this way. Many other cases, however, progressed, became follicular, then ulcerative and passed through the stages above described. It is in this latter class of cases that the medical man and the patient is tried to the uttermost of his skill, patience and perseverance. The treatment of these cases in the field and in western and southern hopitals owes its want of success largely to the fact, that the patients were continually exposed to the exciting causes of the disease.

Removal to northern hospitals, thus securing change of air and surroundings, proper diet and a sparing and judicious use of medicine, saved thousands of lives. The value of medicine in the treatment of these diseases was, by a large number of medical officers of limited experienoe, over-estimated. Many patients in the field, and probably in some of the hospitals, had run the rounds of opium astringents, mercurials, bismuth, strychnia, back to opium, which was the great alleviator of their distress, but could not cure. The experience of our late war has proven to the medical profession, that the diseases under consideration are best treated by furnishing the patient with warmth, pure air and pure water; cleanliness of person, a proper and nutritious diet, pleasant surroundings, not placing too much reliance upon medicine.

The diet best adapted to these cases consists in fresh eggs, custards, milk, chickens, roast beef, roast or boiled lamb, beef soup and fresh fish. Fresh tomatoes, onions and lettuce, starchy preparations of all kinds, except a small amount of good bread, should be disallowed.

Alcholic stimulants are in many, nearly all bad cases, of much value, undoubtedly many lives have been saved by a proper administration of these stimulants.

Brandy, whiskey or Hungarian wine should be given when so powerful a stimulant is required. I believe the latter, when well borne, the best of the three. Claret wine is a favorite at the south, and is an excellent mild stimulant, and very applicable in the convalescing stage of the diseases.

